# Speckle tracking analysis of the left ventricular anterior wall shows significantly decreased relative radial strain patterns in dystrophin deficient mice after 9 months of age

**DOI:** 10.1371/currents.RRN1273

**Published:** 2012-02-02

**Authors:** Christopher Spurney, Qing Yu, Kannaboyina Nagaraju

**Affiliations:** ^*^Associate Professor of Pediatrics, Division of Pediatric Cardiology, Department of Integrative Systems Biology, George Washington University School of Medicine and Health Sciences, Research Center for Genetic Medicine, Children's National Medical Center, Washington, DC; ^†^Research Associate, Research Center for Genetic Medicine, Children’s National Medical Center, Washington, DC and ^‡^Associate Professor of Integrative Systems Biology and Pediatrics, Department of Integrative Systems Biology, George Washington University School of Medicine and Health Sciences, Research Center for Genetic Medicine, Children's National Medical Center, Washington, DC

## Abstract

Background: Duchenne muscular dystrophy (DMD) is an inherited X-linked disorder with an incidence of 1 in 3,500 male births. Early treatment of DMD cardiomyopathy is under investigation and echocardiographic analysis of strain patterns may provide measures to better quantify early treatment outcomes.

Methods: We compared cardiac function in 3, 9 and 12 month old dystrophin deficient mdx mice to wild type (C57BL10/J) using in vivo high frequency echocardiography (Vevo 770, VisualSonics, Inc., Toronto, CA) and 2D speckle tracking [Velocity Vector Imaging (VVI), Siemens Medical Solutions, Inc., Malvern, PA]. Mice were anesthetized with 1-2% inhaled isoflurane and images were obtained using a 30 MHz transducer in modified parasternal long and short axis views obtained at the level of the papillary muscles. Myocardial motion was analyzed using VVI in single-beat reconstructed images.

Results: M-mode imaging showed significantly decreased shortening fraction in mdx mice compared to wild type at 12 months of age (SF% 26.6±3 vs. 32.2±2; p=0.002). Mdx mice showed significantly increased cardiac fibrosis at 12 months of age compared to controls (p<0.0001). Speckle tracking analysis of the left anterior mid ventricular wall segment showed significantly decreased relative radial strain in mdx mice at 9 and 12 months (4.5±1.3% vs. 8.4±0.7%; p=0.001). There were no significant differences in circumferential or longitudinal strain.

Conclusion: Mdx mice show significantly decreased LV anterior mid wall radial strain with mild cardiomyopathy after 9 months of age compared to wild type. Speckle tracking analysis may provide novel outcome measures for preclinical cardiac drug treatment studies in DMD.

##  Introduction:

            Duchenne muscular dystrophy (DMD) is the most common and severe form of muscular dystrophies and occurs in 1 in 3500 male births. Prolonged survival due to improvements in clinical care of the musculoskeletal and respiratory systems has lead to an increased incidence of cardiomyopathy. Cardiac related deaths are now seen in approximately 20% of DMD patients.[1] Early treatment of DMD patients with cardiac medications prior to the development of cardiomyopathy shows promise, but further studies, including preclinical models, are needed.[2] [3] These studies require outcome measures sensitive to changes in myocardial function prior to the development of cardiomyopathy. 

            Speckle tracking imaging (STI) is a new technique which analyzes motion of acoustic reflections inherent in 2D echo images. Now widely accepted in clinical applications, speckle tracking is becoming a validated outcome marker in small animal models. Popovic et al. (2007) performed speckle tracking in a rat myocardial infarction model using parasternal short axis images obtained on a clinical echocardiography platform with a 14 MHz probe. They showed that STI was able to identify segmental left ventricular dysfunction secondary to ischemia.[4] Li et al. (2007) and Luo et al. (2007) performed STI on mouse models of myocardial infarction using a research based platform and a 30 MHz probe, the same used in this study.[5] [6] Peng et al. (2009) performed STI in mouse aortic constriction models using a clinical platform and 14 MHz probe with similar results.[7] STI was used to detect global and region differences in myocardial strain in a mouse myocardial infarction model using a new high frequency echocardiography research platform. Most importantly, the authors also showed improvements in strain measures after treatment with an ACE inhibitor before any changes were seen in shortening fraction.[8] Takano et al. (2011) recently preformed STI in the dystrophin deficient beagle model. They found significantly decreased strain rate in the posterior segments of the left ventricles of carrier and affected dogs compared to controls. All dogs had no significant differences in systolic function.[9] These studies show that speckle tracking and myocardial strain are becoming important preclinical outcome measures for early cardiac dysfunction in small animal models.

            In this study, high frequency based STI was used in the dystrophin deficient mdx mouse model both pre- and post-development of mild cardiomyopathy. We hypothesize that 9 and 12 months old dystrophin deficient mice will have decreased radial strain values compared to controls. Radial strain using STI is a novel outcome measure that may aid in the preclinical evaluation of early drug treatments in DMD cardiomyopathy.

## Methods:

### Animal care: 

All mice were handled according to the Institutional Animal Care and Use Committee guidelines (protocol #01002). Female C57BL/10ScSn-Dmd*^mdx^*/J (*mdx*) and C57BL/10ScSn (wild type) mice weighing 20-25 grams were purchased from the Jackson Laboratory (Bar Harbor, Ma) at 3 months of age and followed longitudinally. All mice were housed in an individually vented cage system with a 12 hour light-dark cycle and received standard mouse chow and purified water *ad libitum*.

### Echocardiography: 

Mice were anesthetized with 1-2% inhaled isoflurane mixed with 100% oxygen as described previously.[10] Two-dimensional, M-mode, and EKV (Electrocardiography-based Kilohertz Visualization) images were obtained using the Vevo 770 (VisualSonics, Inc., Toronto, CA) and a 30 MHz transducer (RMV707B) in modified parasternal long and short axis views obtained at the level of the papillary muscles. Frame rate of EKV imaging is approximately 1000 Hz. Myocardial motion was analyzed using 2D speckle tracking [Velocity Vector Imaging (VVI), Siemens Medical Solutions, Inc., Malvern, PA] on single-beat EKV reconstructed images. For parasternal long axis, the entire myocardium from epicardial to endocardial border was traced and corrected to optimize tracking after processing. Six cardiac segments were measured. For parasternal short axis, the entire myocardium from the epicardial to endocardial border was utilized and 4 cardiac segments were measured, excluding the interventricular septum. Papillary muscles were excluded from endocardial tracings in both views. Speckle tracking images were measured twice by a single investigator (C.S.) blinded to mouse strain and the average was used in the analysis. Images where endocardial or epicardial tracking was deemed inadequate were not analyzed and lead to variations in subject numbers for each age and measurement.

### Collagen quantification (percent fibrosis): 

Five paraffin sections of cardiac tissue (7 um thickness) were stained with picrosirius red (Histoserv, Gaithersburg, Md). The tissue was magnified under a light microscope at an objective of 4X and a digital image of the entire section was obtained using computer software (Olympus C.A.S.T. Stereology System, Olympus America Inc., Center Valley, PA). These digital images were processed using Image J (NIH) with additional threshold color plug-ins to process jpeg images. Pixels corresponding to the area stained in red were normalized to the total pixel area of the tissue image and the results were expressed as percent collagen/ fibrosis. Images were analyzed by two independent, blinded investigators.

### Statistical Analysis: 

For analysis of high frequency echo parameters and percent fibrosis, mean comparisons between strains at each time point used a student’s t-test. Comparisons over time in each strain used a nonparametric trend test. The significance level for each analysis was adjusted for multiple testing using the Bonferroni method. At each time point, comparisons of primary measurement (shortening fraction %) were considered significant at the 0.017 level (1 measurement at 3 time points tested) and secondary measurements at the 0.002 level (9 measurements at 3 time points tested). Across ages, comparisons of primary measurement were considered significant at the 0.05 level (1 measurement tested) and secondary measurements at the 0.006 level (9 measurements tested). For analysis of strain measures, comparisons of primary measurements (anterior wall measures) were considered significant at the 0.004 level (4 measurements at 3 time points tested). Across ages, comparisons of primary measurements were considered significant at the 0.013 level (4 measurements tested). 

## Results:

### High frequency echocardiography: 

Using M-mode imaging, shortening fraction showed mild, but significantly decreased systolic function in mdx mice compared to wild type at 12 months of age (p=0.0016). Mdx mice also showed decreased shortening fraction at 9 months of age compared to wild type that was nearly significant after p values were corrected for multiple testing. (Table 1) When comparing shortening fraction across ages, mdx mice showed a significant decrease in shortening fraction over time (p=0.007) and wild type mice did not (p=0.11).

            Looking at other measures of cardiac size and function using 2D and M-mode imaging, mdx mice showed a decrease in left ventricular internal diameter during diastole at 9 months and a significant decrease when adjusted for multiple testing compared to wild type at 12 months of age (p=0.002). (Table 1) Across ages, mdx mice showed a significant decrease in endocardial percent ejection fraction (p=0.004).

Table 1: Primary (Shortening Fraction %) and secondary echocardiography measurements and percent fibrosis show significantly decreased cardiac function and size in mdx mice and increased fibrosis compared to wild type at 12 months of age. 

**Table:** 

**Measurement**	**Time**	**BL10**		**MDX**		p-value*
		**N**	**Mean ± SD**	**N**	**Mean ± SD**	
Shortening Fraction %	3 months	8	30.5 ± 1.0	8	31.1 ± 3.7	0.6328
	9 months	5	32.4 ± 3.2	5	27.4 ± 2.5	0.0243
	12 months	5	32.2 ± 2.0	8	26.6 ± 2.5	0.0016
Heart Rate (bpm)	3 months	8	464 ± 52	8	445 ± 25	0.3586
	9 months	5	405 ± 34	5	394 ± 45	0.6825
	12 months	5	486 ± 65	8	443 ± 61	0.2535
LV Internal Diameter (d) (mm)	3 months	8	3.88 ± 0.2	8	3.81 ± 0.2	0.5792
	9 months	5	4.08 ± 0.3	5	3.54 ± 0.2	0.0097
	12 months	5	4.24 ± 0.3	8	3.64 ± 0.3	0.0020
LV Posterior Wall (d) (mm)	3 months	8	0.66 ± 0.1	8	0.73 ± 0.1	0.1179
	9 months	5	0.71 ± 0.1	5	0.89 ± 0.2	0.0881
	12 months	5	0.78 ± 0.1	8	0.75 ± 0.1	0.4878
LV mass (mg)	3 months	8	98.6 ± 10.2	8	88.3 ± 14.9	0.1283
	9 months	5	85.3 ± 2.1	5	85.8 ± 8.9	0.9170
	12 months	5	86.4 ± 12.5	8	82.2 ± 11.4	0.5495
Endocardial Ejection Fraction %	3 months	8	52.8 ± 3.0	8	54.3 ± 6.4	0.5562
	9 months	5	55.7 ± 7.8	5	45.3 ± 7.1	0.0588
	12 months	5	51.8 ± 6.3	8	43.8 ± 5.1	0.0275
Myocardial performance index (MPI)	3 months	8	0.50 ± 0.1	8	0.53 ± 0.2	0.7409
	9 months	5	0.55 ± 0.1	5	0.59 ± 0.1	0.5271
	12 months	5	0.64 ± 0.1	8	0.66 ± 0.1	0.6261
Percent Fibrosis	3 months	7	0.3 ± 0.2	7	0.4 ± 0.2	0.6945
	12 months	5	0.7 ± 0.3	8	4.8 ± 0.9	<0.0001

 *Adjusted for multiple testing: significant p value≤0.017 for shortening fraction and p≤0.002 for all other measures; Bpm – beats per minute, LV – left ventricular, d – diastole, mm – millimeters, mg – milligrams

### Percent fibrosis: 

Mdx mice showed a significant increase in percent fibrosis of cardiac tissue at 12 months of age compared to wild type mice (p<0.0001). There was no significant difference in percent fibrosis between mdx and wild type mice at 3 months of age. (Table 1; Figure 1)

**Figure:**
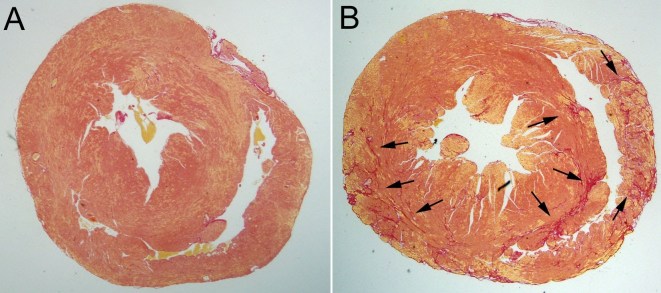
Figure 1: Sirius red stained ventricular tissue from 12 month old BL10 and mdx mice showing significantly increased diffuse fibrosis (areas of dark red staining indicated by arrows) in mdx mice (p<0.0001). The BL10 image corresponds to 0.7% fibrosis and the mdx image is 6.4% fibrosis.

### Myocardial strain analysis: 

Speckle tracking analysis of the anterior mid left ventricular wall segment in EKV modified parasternal short axis images showed significantly decreased radial strain in mdx mice compared to wild type at 9 and 12 months of age (p<0.001 and p<0.01; Table 2 and Figure 2). There were no significant differences in anterior mid left ventricular wall strain at 3 months of age between mdx and wild type mice (p=0.97).

            There were increases in longitudinal strain measures in mdx mice in the EKV modified parasternal long axis images of the anterior base and mid walls. These increases did not reach significant when adjusted for multiple testing (Table 2). 

Table 2: Primary measurements using speckle tracking analysis of the anterior wall in mdx mice compared to wild type shows significantly decreased radial strain at 9 and 12 months of age.

**Table:** 

**Primary Measurement**	**Time**	**BL10**		**MDX**		p-value*
		**N**	**Mean% ± SD**	**N**	**Mean% ± SD**	
PLAX longitudinal anterior base	3 months	8	-4.9 ± 1.1	8	-5.3 ± 2.9	0.7724
	9 months	5	-5.1 ± 1.4	5	-7.7 ± 1.9	0.0348
	12 months	3	-5.8 ± 2.2	4	-7.2 ± 0.6	0.2533
PLAX longitudinal anterior mid	3 months	8	-3.4 ± 1.3	8	-5.3 ± 1.3	0.0092
	9 months	5	-4.1 ± 1.1	5	-4.3 ± 0.8	0.6942
	12 months	3	-2.9 ± 0.3	4	-4.7 ± 1.7	0.1407
PSAX circumferential anterior mid	3 months	8	-5.0 ± 1.8	8	-6.5 ± 1.4	0.0864
	9 months	4	-6.0 ± 1.7	5	-6.4 ± 1.9	0.7748
	12 months	6	-3.6 ± 1.4	6	-4.3 ± 1.6	0.4485
PSAX radial anterior mid	3 months	6	9.2 ± 2.1	8	9.2 ± 2.1	0.9708
	9 months	4	10.0 ± 1.1	5	4.3 ± 1.5	0.0005
	12 months	3	8.4 ± 0.72	7	4.5 ± 1.3	0.0011

*Adjusted for multiple testing: significant p value≤0.004; PLAX – modified parasternal long axis, PSAX – modified parasternal short axis

**Figure:**
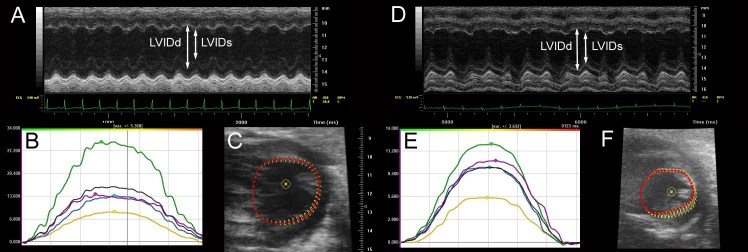
Figure 2: Representative images of M-mode recordings and strain curves showing decreased anterior mid wall strain rates in mdx mice. A) M-mode image of a 12 month old BL10 mouse with arrows indicating the left ventricular internal diameter in diastole (LVIDd) and systole (LVIDs). These measures were used to calculate a shortening fraction of 33% in this image. B) Radial strain curves obtained after analysis of parasternal short axis. The yellow curve corresponds to the anterior mid left ventricular wall and corresponds to a radial strain of 9.14%. C) Reconstructed EKV image of parasternal short axis of a 12 month old BL10 mouse with the endocardial border tracking displayed by yellow circles. The poor tracking of the interventricular septum is noted. D) M-mode image of a 12 month old mdx mouse with LVIDd and LVIDs measures corresponding to a shortening fraction of 23%. B) Radial strain curves obtained after analysis of parasternal short axis. The yellow curve corresponds to the anterior mid left ventricular wall and corresponds to a radial strain of 3.54%. C) Reconstructed EKV image of parasternal short axis of a 12 month old mdx mouse with the endocardial border tracking displayed by yellow circles. The poor tracking of the interventricular septum is again noted.

## Discussion:

            This study shows significantly decreased relative radial strain at 9 and 12 months of age in the anterior mid wall of the left ventricular of dystrophin deficient mice with evidence of mild cardiomyopathy and myocardial fibrosis. These results demonstrate that STI can be utilized in the dystrophin deficient mouse model to provide non-invasive echo based outcome measures for preclinical studies.

            Previous studies also demonstrated decreased shortening fraction in mdx mice compared to wild type at 9 months of age. Similar results for other echocardiographic parameters were also shown.[10] [11] [12] [13] These studies show an average change of 21% in shortening fraction. This study showed a 16-19% change in shortening fraction at 9 and 12 months of age. However, this study showed on average a 47-57% change in radial strain at those same ages. Therefore, radial strain is an outcome measure with the potential to show differences prior to significant changes in shortening fraction resulting from drug therapies in the mdx mouse model.

            Li et al. (2007) studied strain patterns in 2-3 month old mice using similar EKV-reconstructed 2D images, however strain was calculated using 2D minimum sum of absolute differences block matching algorithm. Although the authors report similar difficulties with image signal dropout and rib reverberations, their radial strain values were significantly higher than this study. The maximum anterolateral strain was 45%. This decreased significantly after coronary ligation to 4%.[5] The levels obtained in this study were 9% at 3 months of age. This discrepancy could be related to a third party clinical platform software using EKV reconstructed images. However, even though the magnitude is not the same, the software was able to show relative decreases in mdx mice. Li et al. (2007) also correlated the speckle tracking strain measures with magnetic resonance imaging derived values. Using their technique, strong correlations were demonstrated for both radial (r=0.90) and circumferential (r=0.85) strain in the short axis.[5]

            Pirat et al. (2008) studied strain in a canine model of ischemia using the same software as this study with images obtained using an Acuson clinical platform. Baseline measurements for circumferential strain were -7% and longitudinal strain were -6% in the non-ischemic segment. In this mouse study, 3 month old circumferential strain was -5 to -6.5 and longitudinal strain was -5%. These rates are quite similar for different animal models and different image acquisition platforms.[14]

            In a recent study by Bauer et al. (2011), STI was used to study a mouse myocardial infarction model.[8] The echocardiography system used by Bauer et al. (2011) was the Vevo 2100, manufactured by VisualSonics, Inc. The Vevo2100 uses a new linear-array probe (18 to 38 MHz) and proprietary speckle tracking software (VevoStrain) based in 2D imaging. Bauer et al. (2011) reported a global radial strain value of 28-32% in wild type C57BL6 sham mice. This study did not measure global strain. Bauer et al. (2011) also reported regional longitudinal strain as -11 to -12%. This study found longitudinal strain rates in the anterior wall of -5 to -7%. These decreased levels may again be related to software interpretation of EKV images. Bauer et al. (2011) also reported a decreased radial strain in the infarct zone 3 weeks post infarction. In our model, with evidence of cardiomyopathy at 12 months of age, the anterior wall radial strain in mdx mice is significantly decreased. While the absolute values differ, both studies demonstrate the ability of speckle tracking to utilize strain as an outcome measure in disease models.

            This study demonstrates myocardial strain patterns seen in previous clinical studies of DMD patients. Mori et al. (2007) showed significantly decreased peak systolic radial strain in the posterior wall compared to controls.[15] Ogata et al. (2007) also showed abnormal strain profiles in the posterolateral wall of the left ventricle in DMD patients with normal systolic function.[16] Mertens et al. (2008) demonstrated significantly decreased longitudinal and radial tissue velocities in the anterolateral and inferolateral left ventricular walls in DMD patients (mean age 7.9 years) with normal systolic function.[17] Due to the positioning of the mouse heart during imaging, we were not able to obtain optimal tracking on the lateral left ventricular wall. This area may also become a potential outcome measure using the newer imaging platform.

            Speckle tracking is currently used clinically to evaluate wall motion abnormalities in myocardial ischemia, acute cardiomyopathies and heart failure patients undergoing biventricular pacing.[18] [19] [20] It also has shown promise in the assessment of myocardial viability, diastolic dysfunction and right ventricular mechanics.[18] [21] [22]

### Limitations:

            The current study and methodology demonstrate significant limitations. In order to image the naturally occurring cardiomyopathy, 9 month to 12 month old mdx mice are required. At this age, these mice develop significant static reverberations from the bony sternum and decreased far field resolution that makes automated tracking difficult. In both the long and short axis, this artifact affects the posterior left ventricular wall and apex. Due to this, data from these wall sections were not reliable and not included. Also, although the anterior wall segment demonstrated the best 2D resolution, not all images showed appropriate tracking in this segment. Mice with poor tracking were not included in the analysis, causing group numbers to vary. This is a significant limitation that would need to be compensated for with larger group numbers in subsequent studies, increasing study costs.

            Also, the values obtained for the anterior wall segments are significantly decreased from other mouse studies utilizing younger mice. Our analysis was also based on reconstructed EKV images that can develop some stitching artifact. We also used third party software not specifically developed to handle single loop EKV images and this may account for the decreased strain values. EKV images are reconstructed serial M-mode acquisitions in the direction of radial strain and this may have even more profound effects on measuring longitudinal and circumferential strain. Other studies using the same software showed less variation, however this remains a significant concern for future utilization of this technique. Newer high frequency echocardiography platforms no longer use EKV imaging and include integrated strain evaluation software. Further studies are required to see the magnitude of strain measurements, especially in older mdx mice.

                        In conclusion, mdx mice show significantly decreased relative LV anterior mid wall radial strain with mild cardiomyopathy at 9 and 12 months of age compared to wild type. Speckle tracking analysis of left ventricular anterior wall radial strain and other strain parameters in mdx mice may provide novel outcome measures for preclinical cardiac drug treatment studies, but further optimization of methodology and studies are needed.         

## **Acknowledgments**

None

## **Funding information**

This work was supported with funding from the Muscular Dystrophy Association (MDA 158545 - Spurney), CNMC Clinical Translational Science Institute (Spurney), Department of Defense (W81XWH-09-1-0599 – Nagaraju, Spurney), NCMRR-DC Core Molecular and Functional Outcome Measures in Rehabilitation Medicine (2R24HD050846-06 – Nagaraju), Department of Defense Translational Research for Muscular Dystrophy (10131008 – Nagaraju) and Department of Defense (09166007 – Nagaraju).   

## **Competing interests**

The authors have declared that no competing interests exist. 
